# Intractable Belching as a Psychiatric Consequence: A Case Report

**DOI:** 10.7759/cureus.70191

**Published:** 2024-09-25

**Authors:** Iva Hu, Rachel Alef

**Affiliations:** 1 Psychiatry, Hospital Corporation of America (HCA) Healthcare, Tamarac, USA; 2 Osteopathic Medicine, Nova Southeastern University Dr. Kiran C. Patel College of Osteopathic Medicine, Davie, USA

**Keywords:** belching, cognitive behavioral therapy, psychiatry, psychotherapy, supragastric belching

## Abstract

Background: Belching is a common physiological occurrence, but it becomes bothersome when it occurs excessively and disrupts one’s quality of life. It is hypothesized that psychiatric diagnoses may be an etiology and exacerbation for intractable belching.

Case presentation: We present a case report of a female in her 70s with a psychiatric history of major depressive disorder (MDD) and generalized anxiety disorder (GAD) with symptoms of intractable belching. This patient was diagnosed with supragastric belching (SGB), which is likely behaviorally induced as a result of her psychiatric comorbidities.

Conclusions: In patients presenting with intractable belching, along with other intractable somatic symptoms, psychiatric comorbidities should be considered as a possible etiology or contributing factor. Especially with known psychiatric comorbidities, patients should have maximized psychiatric treatment (including pharmacotherapy and cognitive behavioral therapy) in order to be managed most effectively.

## Introduction

Supragastric belching (SGB) is a type of belching that may begin as a voluntary behavior to relieve symptoms of abdominal discomfort but then later becomes excessive and uncontrolled [1,2]. It is found to be associated with other disorders including, but not limited to, gastroesophageal reflux disease (GERD), obsessive- compulsive disorder (OCD), generalized anxiety disorder (GAD), and major depressive disorder (MDD) [2,3]. It is possible that psychiatric illnesses could indirectly cause supragastric belching. In a study conducted by Punkkinen et al., 26 patients who followed up after treatment for SGB were found to have associated symptoms of anxiety (39% of patients) and depression (26% of patients), as indicated by their scores on the Beck Anxiety Inventory (BAI) and the Beck Depression Inventory (BDI) [4]. It is possible that other undiagnosed psychiatric illnesses may exacerbate or cause intractable SGB. These other psychiatric illnesses may include, but are not limited to, OCD, tic disorders, and somatic symptom disorder. We present a case report of intractable belching in a female in her 70s with a past psychiatric history of MDD and GAD who has been diagnosed with SGB. It may be possible that this patient has underlying psychiatric conditions, in addition to her anxiety and depression, exacerbating her intractable SGB.

## Case presentation

A female in her early 70s with a past psychiatric history of MDD and GAD presented as a new patient to the behavioral health clinic for concerns of depression and generalized anxiety. She endorsed symptoms of anhedonia, amotivation, poor energy, poor appetite, helplessness, poor sleep, and poor focus. The patient described her anxiety as “heavy” feeling. She endorsed feeling anxious about being alone, her physical health, and about what food she would be consuming on a daily basis. She denied a history of manic symptoms, symptoms of panic attacks, psychosis, or post-traumatic stress disorder (PTSD). She reported experiencing belching for “years” and has had associated nausea and vomiting. She cannot control the belching. She denied belching in her sleep. She also reported that she cannot stop her belching for long. Each belch was described as a long, drawn-out, deep belch that would last for about two seconds each. They occur as often as twice a minute.

This patient has no prior psychiatric hospitalizations, suicide attempts, or self-injurious behavior. Medications prescribed at her first psychiatric appointment included sertraline [75 milligrams (mg) daily] for depression and anxiety, clonazepam (0.5 mg, two times a day, as needed) for anxiety, and mirtazapine (15 mg) for sleep and as a mood adjunct at bedtime. Psychosocially, she denied any substance use. She is married with supportive adult children. She is retired. 

Her past medical history includes coronary artery disease with stent placement, valvular disease, uterine cancer, hypertension, and hyperlipidemia. The non-psychiatric medications that she was taking at presentation to her appointment included amlodipine (10 mg daily), hydrochlorothiazide (12.5 mg daily), potassium chloride (20 milliequivalent daily), clopidogrel (75 mg daily), fish oil capsule (1000 mg daily), metoclopramide (10 mg, three times a day), and ondansetron (4 mg as needed for nausea or vomiting). She also has a surgical history of a total hysterectomy.

For the next two years in our clinic, she experienced fluctuating severity of depression and anxiety. She consistently struggled with poor sleep. Her belching tended to worsen with increased anxiety, which was accompanied by nausea, vomiting, and weight loss in varying severity. She was trialed on sertraline and fluoxetine, but both medications were not increased to the maximum dose due to medication side effects such as feeling dull and concern for worsening anxiety due to the medication. She was trialed on haloperidol (2 mg, two times a day) for a potential tic disorder with no improvement, and she ended up being discontinued from it because it made her feel “bad.” She was on haloperidol for one month. She trialed mirtazapine, trazodone, and quetiapine for sleep, but only quetiapine seemed to be effective. At a recent gastroenterology appointment, she was diagnosed with supragastric belching after an extensive workup. 

The patient is currently being treated with the medications quetiapine (100 mg), buspirone (5 mg two times a day), and doxepin (5 mg) to manage her psychiatric illnesses, and she was recommended psychotherapy to assist in her psychiatric comorbidities that may be- and are likely- associated with her SGB diagnosis.

## Discussion

When diagnosing SGB, it is important to exclude other non-behavioral causes of belching in order to manage it most effectively. SGB may be a learned behavior that occurs when there is diaphragmatic anterograde movement and increased pressure in the esophagogastric junction leading to relaxation in the upper esophageal sphincter and expulsion of air out of the esophagus [1,2]. Meanwhile, gastric belching is non-behavioral; physiologically, pressure is not increased in the esophagogastric junction, and instead lower esophageal sphincter relaxation occurs under vagal control [2,5]. While SGB originates from the esophagus, gastric belching originates from the stomach. The two can be distinguished via impedance monitoring [6]. SGB becomes pathological when it presents greater than three days per week and is bothersome to the patient [2]. SGB decreases significantly at night during sleep and when patients are unaware that they are being observed, further indicating the likelihood of behavioral pathogenesis [7,8]. 

There are a variety of psychiatric comorbidities to consider when diagnosing etiologies and/or exacerbating factors of intractable SGB. OCD can be associated with belching and is defined as varying intrusive obsessions and repetitive compulsions performed in an effort to cease obsessions [3,9]. If a patient has continuous obsessions about their learned belching behavior, they may need to continually belch in order to relieve their obsession. For this individual, belching could be seen as an unwanted compulsion of hers. However, she had no reported obsessions, which makes OCD less likely. Belching may also possibly present as a tic, such as persistent vocal tic disorder, which is characterized by one or more vocal tics [10]. The mechanical action and sound of belching may be a vocal tic associated with SGB, especially since the patient reports the belch as something she cannot stop for long and does not continue when she is sleeping, highlighting a functional component. However, tics often develop in childhood, which is not the case for this patient, and a trial of antipsychotics was inefficacious, making a primary tic disorder less likely [10]. Somatic symptom disorder (SSD) may also be a likely comorbid diagnosis with SGB. It is diagnosed based on a significantly bothersome somatic symptom and excessive behaviors or thoughts about the symptoms lasting greater than six months [11]. It is probable that this patient also has SSD as a diagnosis since she has been worrying about her belching for years. She may be having excessive thoughts about her belching, thereby increasing her anxiety and worsening her symptoms. It was revealed that the patient’s belching did worsen with increased anxiety, implying once again an anxious component to the belching. Based on SGB presenting with these other comorbid psychiatric illnesses, it is important to recognize and consider varying differentials with corresponding treatments in order to best lessen the severity of SGB.

Research shows promising results in the use of cognitive behavioral therapy (CBT) as a treatment option for SGB. In an analysis conducted by Sawada et al., CBT was found to improve quality of life and reduce SGB symptoms by 50% [12]. CBT specifically captures patients’ harmful learned behaviors, and by actively bringing underlying thoughts and behaviors to the forefront, patients are able to actively combat the mentally disruptive and destructive behavioral outcome [13]. Of note, diaphragmatic breathing exercises may be used to implement treatment, as a study conducted by Ong and colleagues found increased quality of life scores in patients with SGB who utilized these exercises [14]. 

In another case report conducted by Nagarale et al., a 44-year-old female who presented with eight months of chronic belching was diagnosed with psychogenic belching, and she had significant improvement in symptoms upon completion of eight psychotherapy sessions, with symptoms mostly managed by her second session [15]. This is comparable to the current patient, as SGB may be a form of psychogenic belching. This patient’s gastroenterologist recommended seeking out behavioral therapy in order to maximize her anxiety management. Since this was recently recommended, the patient has not yet received therapy. Based on current research, though, it is likely that participating in CBT (or another form of behavioral therapy) would have a positive impact on this patient’s symptoms and her quality of life. It is also important to note that since this patient’s diagnosis is likely psychiatric in etiology, all comorbid psychiatric conditions in this patient, and in others presenting similarly, should be managed with the appropriate pharmacological therapy in order to receive maximum benefit from treatment.

## Conclusions

This patient with a past psychiatric history of MDD and GAD was ultimately diagnosed with SGB. Her psychiatric comorbidities may have caused or may have been exacerbating factors to her SGB. Therefore, in order to best treat this patient and similar patients, all known psychiatric comorbidities and differential psychiatric comorbidities that could be contributing to the disordered belching should be explored. Based on the concurrent psychiatric diagnoses, it is important to maximize the treatment regimen for those diagnoses. Having the best possible control of those psychiatric disorders may better impact the outcome of improvement in SGB. Prioritizing therapy as an option for these patients is also imperative. This patient was ultimately recommended to pursue psychotherapy as a first-line treatment for her belching. In the bigger picture, clinicians with patients with various behavioral symptoms should consider the possibility of a psychiatric etiology while also considering the possible need to add various therapy options and psychopharmacological treatments to the patient’s management plan.
